# Perceptions on Own Aging: Comparisons between Young Adults with and without Caregiving Experience

**DOI:** 10.1093/geroni/igab046.3722

**Published:** 2021-12-17

**Authors:** Phoebe Clark, Vanessa Cuppari, Matthew Picchiello, Michiko Iwasaki, Andrew Futterman

**Affiliations:** 1 Loyola University MD, Gaithersburg, Maryland, United States; 2 Loyola University Maryland, Baltimore, Maryland, United States; 3 Washington University in St. Louis, St. Louis, Missouri, United States; 4 Loyola University MD, Department Of Psychology, Maryland, United States; 5 Loyola University MD, Loyola University MD, Maryland, United States

## Abstract

Although informal caregiving for older adults (OAs) can increase knowledge and awareness about one’s own aging (Pope, 2013), it can also negatively impact caregivers’ physical health and emotional wellbeing (AARP & NAC, 2020) and have spillover effects on school, work, and marriage (Dellmann-Jenkins & Blankemeyer, 2009). Despite the recent trend of family caregiving for OAs by young adults (YAs), research about these young caregivers is scarce. The present study focused on YAs’ perceptions on aging. We hypothesized that YAs who provided at least three months of caregiving tasks for OAs would hold more awareness and negative perceptions on their own aging, as measured by a modified version of the Brief Aging Perceptions Questionnaire (Sexton et al., 2014), compared to those who did not. We recruited 234 YAs between the ages of 18 - 40 (Mage = 29.78, SD, age = 4.83; 59% White; 65.4% male) and had them complete a survey via Amazon Mechanical Turks. About one third (32.1%) had caregiving experience. Results of independent t-tests revealed that caregivers scored higher on awareness of aging [t(229) = 6.950., p < .001, d = .865] and negative consequences/control [t(231) = 6.528., p < .001, d = .927]. Scores of positive consequences/control did not differ between the two groups. Our findings indicate the need for psychological interventions designed to help young caregivers integrate their caregiving experiences with less negative aging perceptions. Future research should examine the direct effects of caregiving experience on perceptions of aging between young and middle-aged adults.

